# Whole-exome sequencing reveals potential mechanisms of drug resistance to FGFR3-TACC3 targeted therapy and subsequent drug selection: towards a personalized medicine

**DOI:** 10.1186/s12920-020-00794-x

**Published:** 2020-09-21

**Authors:** Zhou Tong, Cong Yan, Yu-An Dong, Ming Yao, Hangyu Zhang, Lulu Liu, Yi Zheng, Peng Zhao, Yimin Wang, Weijia Fang, Feifei Zhang, Weiqin Jiang

**Affiliations:** 1 Department of Medical Oncology, The First Affiliated Hospital, Zhejiang University School of Medicine, Hangzhou, 310003 China; 2 Department of Medical Oncology, The Fourth Affiliated Hospital, Zhejiang University School of Medicine, Yiwu, 322000 China; 3 OrigiMed, Building 3, 115 Xinjun Huan Rd. Minghang, Shanghai, 201114 China; 4 Department of Urology, The First Affiliated Hospital, Zhejiang University School of Medicine, Hangzhou, 310003 China; 5 Provincial Key Laboratory of Pancreatic Disease, Hangzhou, 310003 China; 6 Shanghai LIDE Biotech Co.LTD, Shanghai, 201203 China

**Keywords:** FGFR3-TACC3, Drug resistance, Whole-exome sequencing, Epigenetic regulation

## Abstract

**Background:**

Drug resistance is a major obstacle to effective cancer therapy. In order to detect the change in tumor genomic states under drug selection pressure, we use next-generation sequencing technology to investigate the underlying potential mechanisms of drug resistance.

**Methods:**

In our study, we presented a bladder cancer patient who had been a bona fide responder to first-line gemcitabine plus cisplatin regimen and second-line pazopanib (tyrosine kinase inhibitor (TKI) for *FGFR3-TACC3* fusion) but finally had disease progression as an ideal case for showing genomic alteration during drug resistance. We applied whole-exome sequencing and ultra-deep target sequencing to the patient pre- and post- pazopanib resistance. Protein-protein interaction (PPI) network and Gene Ontology (GO) analyses were used to analysis protein interactions and genomic alterations. Patient-derived xenograft (PDX) model was built to test drug sensitivity.

**Results:**

Twelve mutations scattered in 12 genes were identified by WES pre- pazopanib resistance, while 63 mutations in 50 genes arose post- pazopanib resistance. PPI network showed proteins from multiple epigenetic regulator families were involved post- pazopanib resistance, including subunits of chromatin remodeler SWI/SNF complex ARID1A/1B and SMARCA4, histone acetylation writers CREBBP, histone methylation writer NSD1 and erasers KDM6A/5A. GO enrichment analysis showed pazopanib resistance genes were prominently tagged for chromatin modification, transcription, as well as gland development, leaving genes with the best adaptive FGFR TKI-coping mechanisms. In addition, significantly elevated tumor mutational burden suggested possible utility of immunotherapy. Intriguingly, PDX model suggested that, sensitivity to original chemotherapy regimen (cisplatin) was restored in patient tumor post-pazopanib.

**Conclusions:**

Epigenetic regulation may play a role in acquired TKI resistance. Our study traced the complete tumor genomic variation course from chemo-resistant but TKI-sensitive to TKI-resistant but chemo-(re) sensitive, revealing the potential complex dynamic drug-driven mechanisms of resistance.

## Background

Traditionally, chemotherapy has been an effective first-line treatment for many cancer types. However, apart from its cytotoxicity, chemotherapy is limited in utility for late-stage patients and resistance sooner or later arises [1]. With the onset of next-generation sequencing (NGS) and precision medicine, targeted and immune therapies have come of age and shown remarkable efficacy, even for chemo-resistant late-stage patients [2, 3]. Both targeted and immune therapies, however, suffer from one fatal block. Patients invariably develop drug resistance, be it to small-molecule inhibitors or to antibodies (the former sometimes very soon), bringing an early end to such therapies. Once drug resistance arises, further treatment options are limited and patient’s prognosis is poor. Thus, understanding the underlying molecular mechanisms of drug-driven resistance is not only critical to the advancement of cancer biology, but also invaluable for the practical management of patient care.

Recently, with the advent of NGS, tumor heterogeneity has been better understood; also, resolutions for drug resistance have been attempted using NGS technology [4, 5]. NGS technology, with its ability of high throughput to assess a patient’s comprehensive genomic alterations in a single assay, has been applied to the analysis of tumor samples pre- and post-drug resistance to reveal drug resistance mechanisms. When cohort size is large, simple statistical methods could be enough to identify recurrent mutations and pinpoint genomic or clinical features of interest. However, when data are scarce, it is important to put the few individual signals we have into context to gain insights that might have evaded single-gene analysis.

Protein-protein interaction (PPI) network analysis offers a powerful and flexible tool to integrate various genomic features as a whole. In a PPI network, nodes denote proteins (or their encoding genes) and edges signal direct physical interactions among proteins [6, 7]. Mathematical network structures correspond to biological entities and network analysis could reveal important mechanistic insights. A network hub, a protein with many interaction partners, usually plays a key role in cellular organization [6]. A node’s clustering coefficient (CC), measuring the likelihood of interaction among its direct neighbors, could indicate its function on the spectrum from highly independent enzymes to tightly knitted complex subunits. Gene ontology (GO) offers a systematic approach to classifying a gene’s biological process, molecular function, and cellular component. GO enrichment analysis provides a powerful diagnostic tool for any set of genes, pre- and post-resistance mutated genes in our case.

In the current study, we used PPI network analysis to unite the very few pieces of isolated information (only one patient and a handful of mutations) pre- and post- drug resistance and successfully mined for distinguishing features, some of which were later confirmed by GO enrichment analysis. By using these tools from systems biology, we have gained new insight into the drug resistance mechanism under drug selection pressure.

In our study, an advanced bladder cancer patient achieved almost complete remission (CR) under gemcitabine and cisplatin combination therapy, but exhibited chemo-resistance to the same regimen upon recurrence. We applied both NGS panel assay and whole-exome sequencing (WES) to the patient and discovered a clinically relevant FGFR3-TACC3 fusion. Multi-target tyrosine kinase inhibitor (TKI) pazopanib was administrated and the patient responded exceptionally well, achieving progression-free survival (PFS) of over 10 months before finally succumbing to pazopanib resistance. WES was also performed on post-resistant tissue, and drug-resistant tissue derived patient-derived xenograft (PDX) model suggested sensitivity to cisplatin was restored. Multiple mutations highlighted the potential role of epigenetic regulation in acquired drug resistance, and significantly increased tumor mutational burden (TMB) hinted at likely effectiveness of immunotherapy. Thus, the patient offered an ideal case for detecting the signals differentiating patient genomic states before and after TKI resistance, and our NGS-based analyses closely matched the treatment process.

## Methods

### Patient tumor samples

Fresh tumor specimens were collected at the time of cystoscopy biopsy pre- and post-pazopanib resistance. Our research was permitted by the Ethic Committee of the First Affiliated Hospital of Zhejiang University and the patient was informed of and gave consent to the research use of tumor tissues. All procedures were complied with the principles laid down in the Declaration of Helsinki. After biopsy, tumor tissue was immediately taken to the laboratory and cut into small pieces for genetic analysis.

### Xenograft model

Fresh bladder tumor specimens resected from the patient after pazopanib resistance were implanted subcutaneously into the flanks of 6-week-old mice. The animal experiment was complied with the National Institutes of Health guide for the care and use of Laboratory animals (NIH Publications No. 8023, revised 1978). Mice were anesthetized by intraperitoneal injection of pentobarbital (40 mg/kg). And grafted tumors were subsequently transplanted from mouse to mouse then maintained the model. Xenograft tumor collected at the exponential growth phase were resected aseptically and used as the source of tumor for subcutaneous implantation. The expansion of tumor specimens for drug screening were performed as previously described [8]. Mice are euthanized via intraperitoneal injection of pentobarbital (40 mg/kg) and followed by cervical dislocation. Cohorts of mice with tumor size of ~ 200 mm^3^ were randomized into 8 treatment groups (6 mice per group): (a) vehicle (control); (b) 5-Fu (Xudong Haipu Pharmaceutical. Co.,Ltd., Shanghai, China; dissolved in saline; 25 mg/kg, intraperitoneal, five times a week); (c) docetaxel (Bide Pharmatech Ltd., Shanghai, China; dissolved in 90%saline+ 5%tween80+ 5%ethyl alcohol; 20 mg/kg, intraperitoneal, once a week); (d) mytomycin (Meilun Biological Technology Co.,Ltd., Dalian, China; dissolved in saline; 3.5 mg/kg, intraperitoneal, once a week); (e) irinotecan (Bide Pharmatech Ltd., Shanghai, China; dissolved in saline; 100 mg/kg, intraperitoneal, once a week); (f) cisplatin (Hansoh Pharmaceutical Group Co., Ltd., Jiangsu, China; dissolved in saline; 5 mg/kg, intraperitoneal, once a week); (g) pazopanib (Bide Pharmatech Ltd., Shanghai, China; 0.5%HPMC+ 0.2%tween 80, 100 mg/kg, p.o. once a day); (h) pemetrexed (Hansoh Pharmaceutical Group Co., Ltd., Jiangsu, China; dissolved in saline; 200 mg/kg, intraperitoneal, once a week). Tumor size was evaluated twice a week by caliper measurements, and tumor volume was calculated using the following formula: tumor volume = [length x width^2] / 2. Mice were euthanized if tumor size reached 1500 ~ 2000 mm^3^ or weight loss was greater than 15% as per Institutional Animal Care and Use Committee (IACUC) protocol. Tumor growth in drug-treated animals was compared to that of control group and represented as percentage tumor growth inhibition (TGI). TGI (%) = [1-(Tt-T0)/Ct-C0]*100, where Tt = median tumor volume of treatment group at time t, T0 = median tumor volume of treatment group at time 0, Ct = median tumor volume of control at time t and C0 = median tumor volume of control at time 0.

### Library preparation and whole-exome sequencing

Paired-end DNA library was prepared according to the manufacturer’s instructions (Agilent). The adapter-modified gDNA fragments were enriched by 6 cycles of PCR. Whole exome capture was carried out using Agilent’s SureSelect Human All Exon V5 Kit. Finally, 50 Mb of DNA sequences of 33,4378 exons from 20,965 genes were captured. After DNA quality evaluation, pooled samples were sequenced on Illunima Hiseq 4000 according to the manufactures instructions for paired-end 150 bp reads. The average sequencing depth of target region was 200X and coverage of target region was 99.8%.

### Exome sequencing data analysis for SNVs and INDELs calling

Raw data (stored as FastQ format) obtained from Hiseq4000 contains adapter contamination, low-quality nucleotide, and undetected nucleotide (N), which can pose significant influence on downstream processing analysis. Hence, reads with adapter contamination, reads containing uncertain nucleotides more than 10 percentages, and paired reads when single reads have more than 50 percentages low-quality (< 5) nucleotides are discarded. After these steps, high-quality clean data are obtained. Finally, QC statistics including total reads number, sequencing error rate, percentage of reads with average quality >Q20, percentage of reads with average quality >Q30, and GC content distribution can be calculated. Paired-end clean reads are aligned to the reference genome (UCSC hg19) using Burrows–Wheeler Aligner (BWA) software. If a read or reads pair is mapped to multiple positions, BWA will choose the most likely placement. While if two or more most-likely placements are present, BWA will choose any one randomly. Aligned reads were realigned to the genome. Genome Analysis Toolkit (GATK) was used to ignore those duplicates resulted from PCR amplification with Picard-tool. We utilized the Indelrealigner and Realigner Target Creator in GATK do realignment around the INDELs according to GATK best practice. Furthermore, we performed base quality score recalibration with GATK to avoid system bias. After realignment to genome, we identified and filtered variants (SNP, INDELs) using GATK Haplotype Caller and variant Filtration to guarantee meaningful analysis. Variants obtained from previous steps were compared based on the dbSNP and 1000 Genomes database and annotates with ANNOVAR. SNVs and somatic INDELs were identified using MuTect and Strelka with matched normal samples, respectively.

### Ultra-deep target sequencing

Fresh tissue sections from the patient were collected and DNA extracted. Paired-end sequencing (2 × 75 bp) was carried out on Illumina NextSeq500 instrument following the manufacturer’s protocols. NGS panel assay was performed against 365 common cancer-related genes (Suppl Tab. 1) and selected frequently rearranged introns. Genomic alterations, including single nucleotide variants (SNVs), short and long insertions/deletions (indels), copy number variations (CNVs), and gene rearrangements, were subjected to advanced analysis. First, reads were aligned to human genome reference sequence (hg19) by Burrows-Wheeler Aligner (BWA), and PCR duplicates were removed using Picard. Secondly, SNVs and short indels were identified by MUTECT after quality recalibration and realignment using GATK and in-house pipeline. Short indels were then calibrated using the results from Pindel. Read depths were normalized within target regions by EXCATOR. The log-ratio per region of each gene was calculated, and customized algorithms were used to detect copy number changes. Tumor cellularity was estimated by allele frequencies of sequenced SNPs. A customized algorithm was developed to detect gene rearrangements and long indels.

Reliable somatic alterations were detected in the raw data by comparison with matched blood control samples. At minimum, 5 reads and minimum variant allele frequency of 1% were required to support alternative calling. For CNVs, focal amplifications were characterized as genes with thresholds ≥4 copies for amplification and 0 copies for homozygous deletions. For the calling of gene rearrangements, aligned reads with abnormal insert size of over 2000 or zero bp were collected and used as discordant reads. Next, the discordant reads with the distance less than 500 bp formed clusters that were further assembled to identify potential rearrangement breakpoints. The breakpoints were reconfirmed by BLAT and the resulted chimeric gene candidates were annotated. Clinically relevant genomic alterations were further marked as druggable genomic alterations in current treatments or clinical trials. The average sequencing depth were 600X for tissue based deep sequencing.

### Tumor mutational burden computation

TMB was estimated by counting somatic mutations including coding single base substitutions and insertions/deletions per megabase of the examined genomic sequence. Driver gene mutations and known germline variants in dbSNP were excluded.

### PPI network analysis

A representative human PPI network was assembled from several large-scale, experimentally derived PPI assays [6, 7, 9]. The single largest connected component of this network consists of 9316 nodes and 42,102 edges. All network analysis was based on this giant component. Binary PPIs were assembled from several large-scale yeast two-hybrid (Y2H) screens: redundant interactions were filtered out, self-interactions were excluded, and interactions were considered un-directional (A-B and B-A counted only once). The single largest connected component of the resulting PPI network was extracted and used for all further analysis. Network parameters such as node degree and clustering coefficient were computed using custom Python and R scripts. Network visualization was done in Cytoscape 3.6.1 (http://www.cytoscape.org). Network simulations were carried out using custom Python and R scripts. Statistical tests were performed using R (version 3.3.2, http://www.r-project.org).

### GO-terms enrichment analysis

GO enrichment analysis was performed using Cytoscape-plugin BiNGO. Mutated genes were compared to the background of all network genes using hypergeometric test with FDR correction. Enriched GO terms were filtered through a stringent threshold of corrected *p*-value < 0.001 and then visualized through hierarchic clustering in Cytoscape.

## Results

### Patient history

**Fig. 1:**
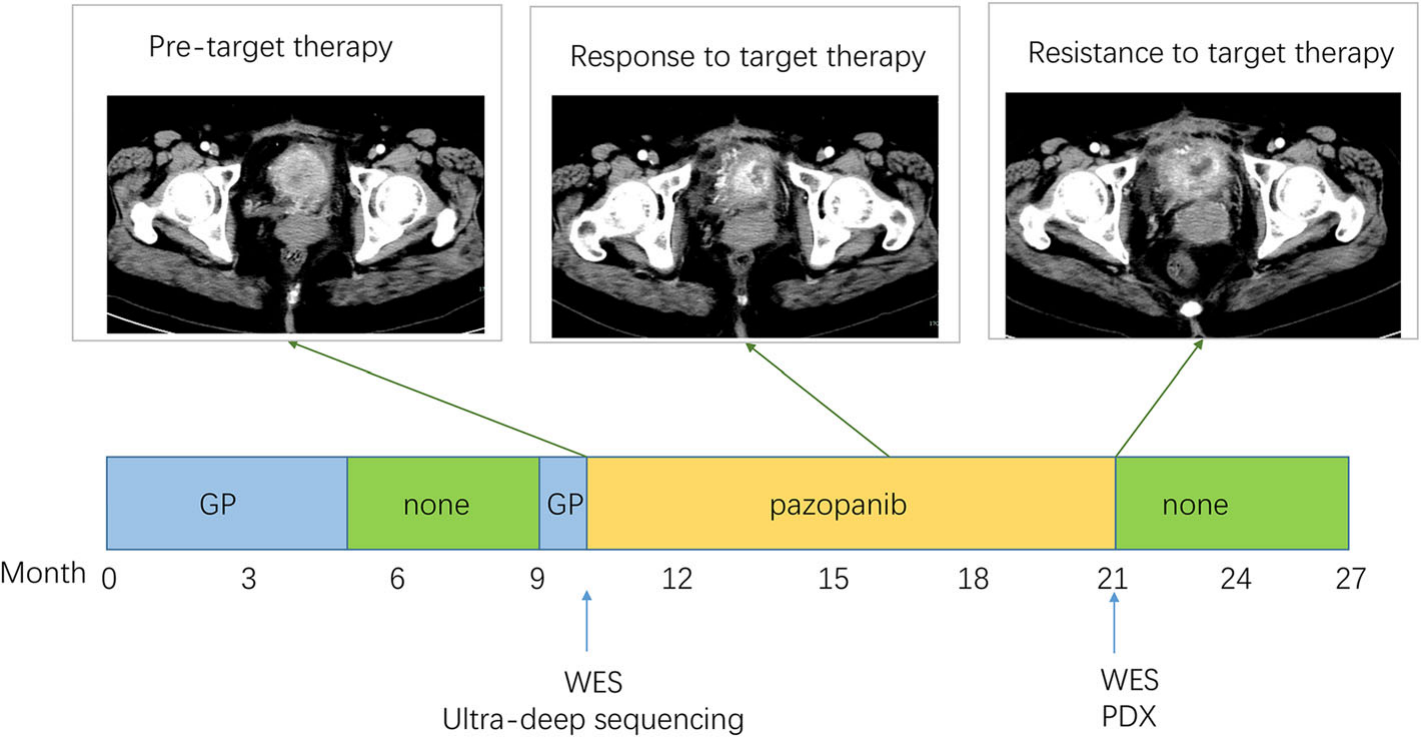
Treatment timeline for the presented patient. The patient’s initial chemotherapy regimen was gemcitabine and cisplatin (GP). After resistance to GP regimen, informed by the results of genetic tests, pazopanib was used as second-line treatment. Abdominal computer tomography showed significantly shrunk tumor size (middle image), and the patient acquired 10 months PFS. Pazopanib naïve tumor tissue were performed for both ultra-deep sequencing and WES, while tumor tissue after pazopanib resistance were performed for WES. Post-pazopanib resistant tissue also were used for PDX model

In October 2014, a 50-year-old woman who presented intermittent gross hematuria received the segmental cystectomy in a local hospital. Postoperative pathology showed high-grade urothelial carcinoma (Suppl Fig. 1c). No chemotherapy was performed after the operation. Five months later, the patient presented gross hematuria again and came to our hospital for further examination. Computed tomography (CT) imaging of the abdomen revealed 4.9*4.7 cm metastatic tumor near left pelvic wall (Suppl Fig. 1a) and Carcinoembryonic antigen (CEA) elevated to 14.2 ng/ml. Physical examination showed no positive sign, and Eastern Cooperative Oncology Group (ECOG) performance status was 0. 6 cycles of gemcitabine and cisplatin was initiated with gemcitabine 1000 mg/m2 during 30 to 60 min on days 1, 8, plus cisplatin 25 mg/m^2^ on day 1–3, Cycles were repeated every 21 days. Follow-up CT and serum tumor marker examinations were performed every 3 months. After 6 cycles of treatment, CT scan showed partial response (PR), almost complete response (CR) of the mass according to the Response Evaluation Criteria in Solid Tumors (RECIST) version 1.1 guideline (Suppl Fig. 1b). The patient repeated CT scan and tumor marker every 3 months and there was no sign of recurrence. However, in January 2016, the patient demonstrated progressive disease (PD) in the bladder with a 4.1*3.7 cm (cm) tumor mass. And CEA elevated to 30.6 ng/ml. After the patient exhibited progressive disease (PD) in the bladder, 2 cycles of gemcitabine and cisplatin regimen were again initiated but was ineffective. In light of resistance to chemotherapy, cystoscopic tissue biopsy was performed and we applied both WES and 365-gene NGS panel assay to this patient and identified a clinically actionable FGFR3-TACC3 fusion mutation. FGFR3 is one of the targets to the multi-target tyrosine kinase inhibitor pazopanib, thus pazopanib was administrated and the patient responded exceptionally well, achieving progression-free survival (PFS) of over 10 months before finally succumbing to drug resistance. Re-biopsy was done through cystoscope after progress of pazopanib, WES was performed on post-resistant tissue (Suppl Fig. 1d), pazopanib resistant tissue derived PDX model was built to test drug sensitivity. The treatment timeline is shown in Fig. 1. A host of new mutations, many implicating epigenetic regulation, arose post drug resistance, and drug screening assay based on PDX model suggested possible re-sensitization to the initial cisplatin regimen.

### Comparison of NGS results

A total of four rounds of NGS sequencing were performed: 365-gene panel assay and WES at the baseline of pazopanib treatment, both using tumor tissue DNA (Panel1 and WES1); WES after pazopanib resistance, using tumor tissue DNA (WES2). Many of the mutations found in the patient were validated. Among all the alterations, only 1 alteration (FGFR3-TACC3) has clinical significance [10, 11], 7 mutated genes (DICER1 M1L, EP300 Q224*, FAM135B L633*, FANCD2 S240*, GATA6 S184N, KDM6A H900Qfs*11, TP53 E258K) have potential clinical significance, 8 mutated genes (ARHGAP26 R719W, ARID1A P153A, ATM A799T, LRP1 V3244I, MLLT3 E231D, NCOR1 T1870N, NRG3 E355D, PLAG1 V18A) are known somatic alterations in COSMIC but have no functional analysis study, others are unknown mutations. Panel1 identified 12 mutations scattered in 12 genes, so did WES1; however, those two sets only partially overlap, reflecting the potential depth-coverage trade-off of the underlying sequencing technologies. On the other hand, WES2 revealed many more mutations, 63 spread across 50 genes. The mutations that stayed the same from Panel1/WES1 to WES2 such as EP 300 Q224*, FAM135B L633*, IGF2 Q7P and TP53 E258K, which were most likely involved in the process of tumorigenesis or chemo-resistance, could be ruled out as contributors to FGFR TKI resistance. On the other hand, new mutations arising post pazopanib resistance warranted further investigation and the difference between pre- and post TKI-resistance was the focus of our study. Finally, the overwhelming majority of mutations arising post drug resistance nearly all had very low VAF (Table 1). We did the copy number alteration and also rearrangement/fusion analysis on data from whole exome sequencing and ultra-deep sequencing. Unfortunately, there were no specific tumor suppress genes or oncogenes involved in the potential mechanism.

**Table 1:** Comparison of genomic alterations pre- and post-pazopanib resistance

Gene Name	Panel1					WES1					WES2					Impact of Mutation
	cDNA change	aa change	mutant reads	coverage	VAF	cDNA change	aa change	mutant reads	coverage	VAF	cDNA change	aa change	mutant reads	coverage	VAF	
FGFR3	NA	FGFR3-TACC3	900	NA	NA	NA	NA	NA	NA	NA	NA	NA	NA	NA	NA	Clinical significance
ACVR1B	NA	NA	NA	NA	NA	NA	NA	NA	NA	NA	c.85G > A	V29I	9	218	0.04	Unknown significance
ADGRA2	c.G3010C	G1004R	43	782	0.06	NA	NA	NA	NA	NA	c.1883C > G	S628C	9	228	0.04	Unknown significance
AKT3	NA	NA	NA	NA	NA	NA	NA	NA	NA	NA	c.432A > G	T144T	8	111	0.07	Unknown significance
ARHGAP26	NA	NA	NA	NA	NA	NA	NA	NA	NA	NA	c.2155C > T	R719W	15	331	0.05	Confirmed somatic
ARID1A	NA	NA	NA	NA	NA	NA	NA	NA	NA	NA	c.352A > C	T118P	11	110	0.10	Unknown significance
											c.780_782delCTC	S261del	16	179	0.09	Unknown significance
											c.1040_1041insAGC	A347dup	13	154	0.08	Unknown significance
											c.457C > G	P153A	9	191	0.05	Confirmed somatic
ARID1B	NA	NA	NA	NA	NA	NA	NA	NA	NA	NA	c.832G > A	G278S	23	272	0.09	Unknown significance
											c.884G > A	C295Y	19	273	0.07	Unknown significance
											c.1437G > A	M479I	7	131	0.05	Unknown significance
											c.1189 T > G	S397A	5	172	0.03	Unknown significance
ATM	NA	NA	NA	NA	NA	c.2395G > A	A799T	13	162	0.08	NA	NA	NA	NA	NA	Confirmed somatic
CDK12	NA	NA	NA	NA	NA	NA	NA	NA	NA	NA	c.1489G > T	A497S	11	172	0.06	Unknown significance
CDK6	NA	NA	NA	NA	NA	c.378G > T	M126I	6	151	0.04	c.378G > T	M126I	8	179	0.05	Unknown significance
CDK8	NA	NA	NA	NA	NA	NA	NA	NA	NA	NA	c.703C > T	H235Y	5	206	0.02	Unknown significance
CHD4	NA	NA	NA	NA	NA	NA	NA	NA	NA	NA	c.226A > T	M76L	6	69	0.09	Unknown significance
CREBBP	NA	NA	NA	NA	NA	NA	NA	NA	NA	NA	c.146_148delGAG	G49del	5	226	0.02	Unknown significance
CSNK1A1	NA	NA	NA	NA	NA	NA	NA	NA	NA	NA	c.505A > C	T169P	5	178	0.03	Unknown significance
CUL3	NA	NA	NA	NA	NA	NA	NA	NA	NA	NA	c.464A > T	D155V	32	98	0.33	Unknown significance
DICER1	NA	NA	NA	NA	NA	NA	NA	NA	NA	NA	c.1A > T	M1L	7	90	0.08	Potential clinical significance
DPYD	NA	NA	NA	NA	NA	NA	NA	NA	NA	NA	c.170A > G	N57S	6	283	0.02	Unknown significance
											c.229A > G	M77V	8	410	0.02	Unknown significance
EP300	c.670C > T	Q224*	628	969	0.65	c.670C > T	Q224*	27	95	0.28	c.670C > T	Q224*	137	143	0.96	Potential clinical significance
EPHA5	NA	NA	NA	NA	NA	NA	NA	NA	NA	NA	c.1803A > T	E601D	5	106	0.05	Unknown significance
ERBB3	c.G2641C	E881Q	155	960	0.16	NA	NA	NA	NA	NA	c.2641G > C	E881Q	46	142	0.32	Unknown significance
ETV1	NA	NA	NA	NA	NA	NA	NA	NA	NA	NA	c.68A > C	N23T	6	146	0.04	Unknown significance
EWSR1	NA	NA	NA	NA	NA	NA	NA	NA	NA	NA	c.898A > T	M300L	6	82	0.07	Unknown significance
											c.988A > C	M330L	6	142	0.04	Unknown significance
EXT1	NA	NA	NA	NA	NA	NA	NA	NA	NA	NA	c.[876 T > C;877G > T]	[V292V;V293L]	12	453	0.03	Unknown significance
FAM135B	c.1898 T > A	L633*	257	975	0.26	c.1898 T > A	L633*	5	144	0.04	c.1898 T > A	L633*	67	318	0.21	Potential clinical significance
FANCD2	NA	NA	NA	NA	NA	NA	NA	NA	NA	NA	c.719C > A	S240*	8	164	0.05	Potential clinical significance
											c.859C > T	H287Y	8	249	0.03	Unknown significance
FAT3	NA	NA	NA	NA	NA	c.6854C > A	P2285H	16	141	0.11	c.6854C > A	P2285H	61	303	0.20	Unknown significance
FLT1	NA	NA	NA	NA	NA	NA	NA	NA	NA	NA	c.2515G > T	G839*	57	289	0.20	Unknown significance
FOXP1	NA	NA	NA	NA	NA	NA	NA	NA	NA	NA	c.1625A > T	K542I	12	150	0.08	Unknown significance
GATA6	c.G551A	S184N	26	338	0.08	NA	NA	NA	NA	NA	NA	NA	NA	NA	NA	Potential clinical significance
GLI2	NA	NA	NA	NA	NA	NA	NA	NA	NA	NA	c.4112C > T	P1371L	6	197	0.03	Unknown significance
HRAS	c.A422C	Y141S	32	626	0.05	NA	NA	NA	NA	NA	NA	NA	NA	NA	NA	Unknown significance
IGF2	c.20A > C	Q7P	94	445	0.21	c.20A > C	Q7P	12	153	0.08	c.20A > C	Q7P	105	217	0.48	Unknown significance
IKZF1	NA	NA	NA	NA	NA	NA	NA	NA	NA	NA	c.346G > C	D116H	6	276	0.02	Unknown significance
KAT6A	NA	NA	NA	NA	NA	NA	NA	NA	NA	NA	c.4769G > T	G1590V	20	349	0.06	Unknown significance
KDM5A	NA	NA	NA	NA	NA	NA	NA	NA	NA	NA	c.396G > A	M132I	10	304	0.03	Unknown significance
KDM6A	c.2700_2724del	H848Qfs*11	499	1026	0.49	c.2700_2724del	H900Qfs*11	46	318	0.15	c.2700_2724del	H900Qfs*11	260	267	0.97	Potential clinical significance
	NA	NA	NA	NA	NA	NA	NA	NA	NA	NA	c.2327 T > C	I776T	12	156	0.08	Unknown significance
	NA	NA	NA	NA	NA	NA	NA	NA	NA	NA	c.1423 T > C	S475P	8	113	0.07	Unknown significance
	NA	NA	NA	NA	NA	NA	NA	NA	NA	NA	c.2038G > A	A680T	9	163	0.06	Unknown significance
LIMK1	NA	NA	NA	NA	NA	c.695G > A	R232Q	7	214	0.03	NA	NA	NA	NA	NA	Unknown significance
LRP1	NA	NA	NA	NA	NA	c.9730G > A	V3244I	5	213	0.02	NA	NA	NA	NA	NA	Confirmed somatic
NCOR1	NA	NA	NA	NA	NA	NA	NA	NA	NA	NA	c.5609C > A	T1870N	14	150	0.09	Confirmed somatic
NF1	NA	NA	NA	NA	NA	NA	NA	NA	NA	NA	c.4154A > G	K1385R	20	234	0.09	Unknown significance
NFIB	NA	NA	NA	NA	NA	NA	NA	NA	NA	NA	c.1346G > C	S449T	13	292	0.05	Unknown significance
NKX2–1	NA	NA	NA	NA	NA	NA	NA	NA	NA	NA	c.16A > G	S6G	5	219	0.02	Unknown significance
NRG3	NA	NA	NA	NA	NA	NA	NA	NA	NA	NA	c.2090G > A	E355D	6	128	0.05	Confirmed somatic
NSD1	NA	NA	NA	NA	NA	NA	NA	NA	NA	NA	c.155_157dupCTG	T52ins	23	273	0.08	Unknown significance
											c.158 T > C	V53A	5	152	0.03	Unknown significance
NTRK3	NA	NA	NA	NA	NA	NA	NA	NA	NA	NA	c.1698A > T	P541S	27	101	0.27	Unknown significance
PLAG1	NA	NA	NA	NA	NA	NA	NA	NA	NA	NA	c.53 T > C	V18A	10	245	0.04	Confirmed somatic
RANBP2	c.G3469C	D1157H	123	832	0.15	NA	NA	NA	NA	NA	c.2591C > A	D1157H	101	276	0.37	Unknown significance
RHOA	NA	NA	NA	NA	NA	NA	NA	NA	NA	NA	c.209G > T	E186K	15	144	0.10	Unknown significance
SMARCA4	NA	NA	NA	NA	NA	NA	NA	NA	NA	NA	c.1930G > A	A1423A	7	277	0.03	Unknown significance
SSX1	NA	NA	NA	NA	NA	NA	NA	NA	NA	NA	c.358G > A	K153N	136	257	0.53	Unknown significance
TCF7L2	NA	NA	NA	NA	NA	c.670G > A	V224I	5	198	0.03	c.670G > A	S467A	13	172	0.08	Unknown significance
TEK	c.C3251A	S1084*	63	474	0.13	NA	NA	NA	NA	NA	c.2932G > A	S1084*	40	129	0.31	Unknown significance
TERT	NA	NA	NA	NA	NA	c.1520A > T	E507V	21	348	0.06	c.2105C > T	E507V	130	645	0.20	Unknown significance
TP53	c.772G > A	E258K	235	460	0.51	c.772G > A	E258K	53	241	0.22	c.31G > A	E258K	121	128	0.95	Potential clinical significance
ZNF703	NA	NA	NA	NA	NA	NA	NA	NA	NA	NA	c.58A > G	G21ins	54	599	0.09	Unknown significance

Note: Clinical significance were variations had been reported as functional variants in cancer samples with functional studies. Potential clinical significance was those predicted to be functional, mainly for those splicing site or truncation of tumor suppressor genes. Confirmed somatic were known somatic alterations in COSMIC but have no functional analysis study. Others were defined as Unknown significance

### TMB value before and after TKI-resistance

The TMB value of tumor tissue increased from 38.4 mut/Mb to 97.2 mut/Mb between the baseline and resistance of pazopanib, suggesting that the patient could potentially further benefit from immunotherapy after acquiring pazopanib resistance. It remains to be seen whether this is a universal feature of developing resistance in bladder and other cancers.

### PPI network and functional analysis of mutations pre- and post pazopanib resistance

**Fig. 2:**
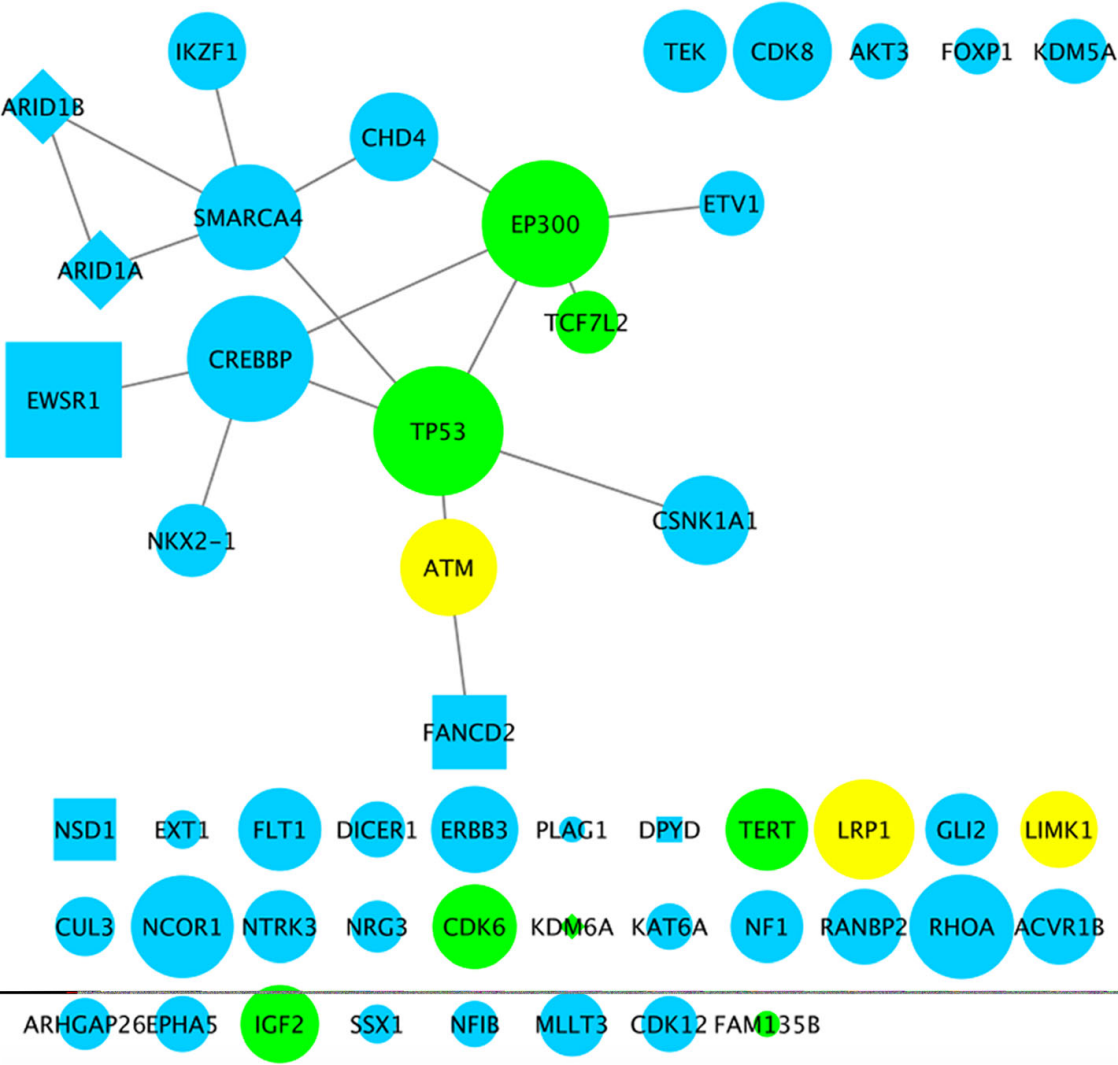
Network of mutated genes pre- and post-pazopanib resistance. Green nodes denote genes that were mutated both before and after resistance; yellow nodes denote genes that were mutated before resistance but not afterwards; blue nodes denote genes that were newly mutated after resistance. Node shape codes for the number of distinct mutations a gene had: circle = 1, square = 2, diamond = 4. Node size corresponds to the number of interaction partners a gene had in the PPI network. Two genes were connected by an edge if their corresponding (canonical) protein products physically interacted in the PPI network

We mapped the mutations onto an experimentally derived human PPI network and extracted the subnetwork of impacted genes (Fig. 2). Strikingly, a tightly woven new cluster arose beside the conserved TP53-EP300 axis. In particular, multiple proteins from multiple epigenetic regulator families were involved, some harboring multiple mutations. Examples include subunits of chromatin remodeler SWI/SNF complex ARID1A/1B and SMARCA4, histone acetylation writers CREBBP, histone methylation writer NSD1 and erasers KDM6A/5A.

### Network structures and simulations

**Fig. 3:**
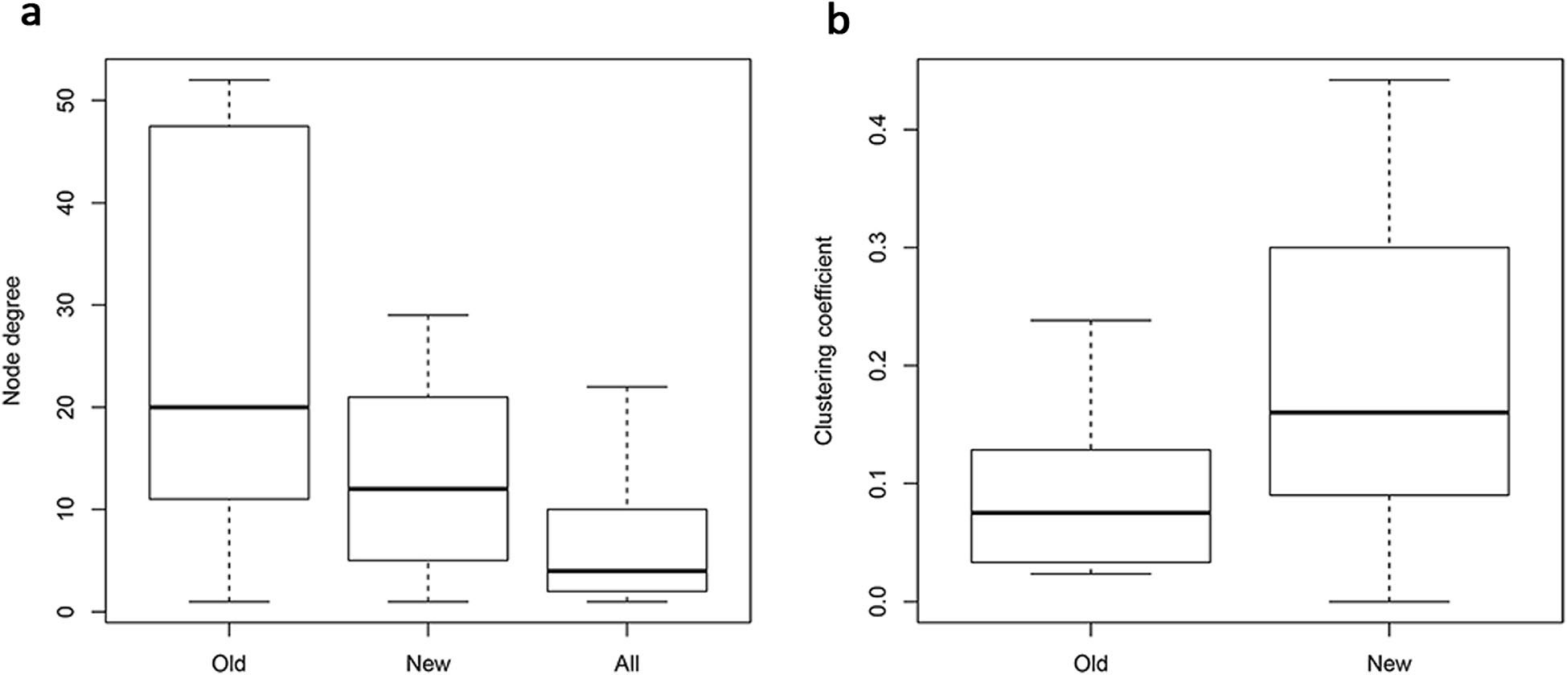
Different hubs in tumorigenesis and drug resistance. Node degree means the number of interaction partners a gene had in the PPI network. “Old” represents the initial set of genes mutated pre-pazopanib resistance; “New” represents the newly mutated genes post-pazopanib resistance; “All” represents all genes in the PPI network as the background for comparison. **a** Newly mutated genes (post-pazopanib resistance) tended to be smaller hubs than the “old” genes(*p* < 0.05). **b** New mutations were more clustered. Clustering coefficient (CC) counted the fraction of realized interactions out of all possible pair-wise interactions among the direct neighbors of a given node. Newly mutated genes post-pazopanib resistance (“New”) had significantly higher CC than those pre-pazopanib resistance (“Old”) (*p* < 0.05)

The initial set of mutated genes (pre- pazopanib resistance) are predominantly network hubs (Fig. 3a), suggesting that such highly connected nodes, already proven critical to many aspects of cell biology, could also play an important role in tumorigenesis. In comparison, newly mutated genes (post pazopanib resistance) tend to be smaller hubs with significantly higher clustering coefficient (CC), hinting at a different mechanism leading to drug resistance **(**Fig. 3a & b). To make sure that our observations were not an artifact of network degree distribution, which has a profound effect on other network parameters, we carried out simulations by picking 10,000 random samples with the same degree distribution as the set of tumorigenesis (or drug resistance, respectively) genes and comparing the background to the observed. The set of tumorigenesis genes identified in pazopanib naïve sample are not only hubs, but also network centers even compared to other hubs (Suppl Fig. 2), highlighting their central cellular roles. In comparison, the set of drug resistance genes identified in pazopanib-resistant sample not only have higher CC, but also possess greater K1 centrality measure (average neighbor degree), suggesting their function coordinating other hubs and processes (Suppl Figs. 3 & 4). It suggested that tumorigenesis genes pre-pazopanib resistance were major network hubs playing central cellular roles, while those genes associated with pazopanib resistance were much more dispersive and could derive their evasiveness through coordinative-interactive effect. Taken together, network analysis revealed distinct features and reflected different drug resistance mechanisms under the different drug selection pressure.

### GO enrichment analysis

**Fig. 4:**
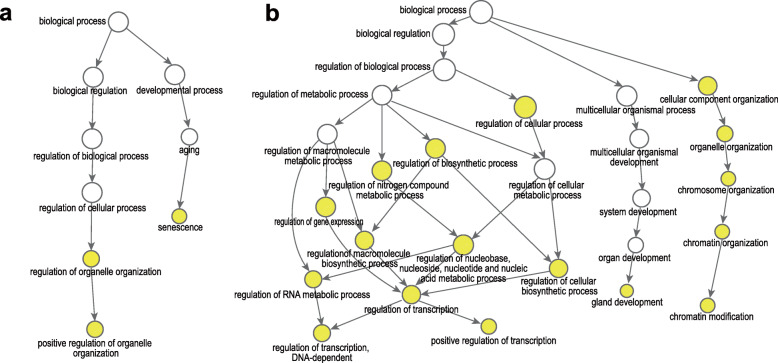
GO enrichment analysis of mutated genes pre- and post-pazopanib resistance. **a** GO enrichment analysis showed enriched biological processes were senescence and regulation of organelle organization pre-resistance. **b** Chromatin modification, regulation of transcription, and gland development were enriched post-resistance. Color gradient corresponded to significance of corrected *p*-values

We performed GO enrichment analysis on the sets of genes mutated before and after pazopanib resistance. Tumorigenesis genes were primarily enriched in senescence and regulation of organelle organization (Fig. 4a). The former function likely reflects tumor’s ability to reverse a normal cell’s aging process and reactivate division and growth, thereby achieving “immortality”. In comparison, drug resistance genes were prominently tagged for chromatin modification and many aspects of transcription, as well as gland development (Fig. 4b**,** Suppl Fig. 5). Again, the data suggested that in face of targeted drug pressure, tumor cells scrambled to react and epigenetic regulation occupied central stage.

### PDX model based drug screening assay

**Fig. 5:**
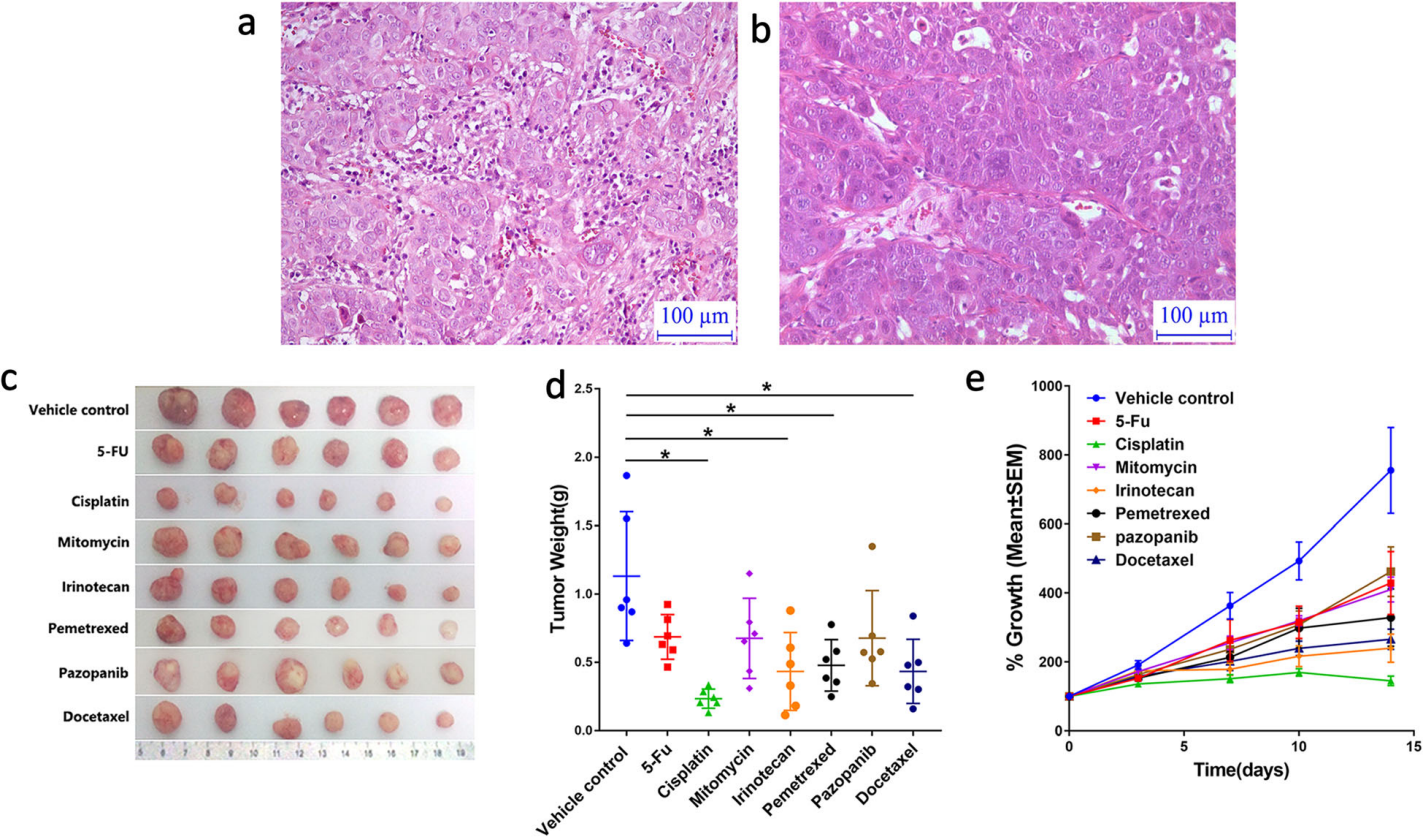
Patient-derived xenograft (PDX) model suggested sensitivity to cisplatin was restored. Hematoxylin and eosin (H&E) staining of patient tumor. **b** Hematoxylin and eosin (H&E) staining of PDX model. **c** Gross view of PDX tumor at the end of the experiment. **d** Tumor weight calculated at the end of the experiment. Average weight of vehicle, cisplatin, docetaxel, irinotecan, 5-Fu, mitomycin, pemetrexed and pazopanib groups were 1.13 ± 0.47 g, 0.23 ± 0.07 g, 0.43 ± 0.23 g, 0.43 ± 0.29 g, 0.69 ± 0.16 g, 0.68 ± 0.29 g, 0.48 ± 0.19 g and 0.68 ± 0.35 g.*P < 0.05. **e** Tumor growth curves of all groups. Treatment groups (TGI > 50%) excluding pazopanib group show statistically significant tumor growth inhibition compared to vehicle group. TGI > 50% is considered meaningful

The objective of the study was to evaluate the safety and anti-cancer utility of the selected drugs on xenograft models in Nu/Nu mice. No major differences were observed between our PDX model and patient tumor in terms of cell and tissue architecture (Fig. 5a, b). Figure 5c shows gross view of PDX tumor at the end of the experiment. Compared to control group, the tumor weight decreased significantly in the cisplatin in the PDX model (Fig. 5d**)**. All groups except pazopanib group showed significant tumor growth inhibition compared with vehicle group (Fig. 5e), which was consistent with our clinical situation that the patient acquired pazopanib resistance. Our data suggested that sensitivity to original chemotherapy regimen (cisplatin) was restored in patient tumor post-pazopanib.

## Discussion

Drug-driven resistance is common, which reflects the complicated course of tumor genomic alteration under the drug pressure. It has been observed and explored in multiple types of cancer, including lung cancer [12], pancreatic cancer [13], colorectal cancer [14] and so on. And researchers tried to find out the deep drug-driven resistance mechanisms. In our study, our patient had failed first-line chemotherapy and second-line FGFR TKI treatment, and finally PDX model showed possible re-sensitization to the initial cisplatin regimen. We described a classic case of dynamic drug-driven resistance in a complicated course of chemo-resistant but TKI-sensitive to TKI-resistant but possible chemo-(re)sensitive.

Many of the mutations found in the patient were validated. The p53 tumor suppressor protein encoded by the TP53 gene is generally functionally deficient in advanced malignant tumors [15]. Mutations in TP53 were observed in about 50% of muscle-invasive bladder cancers but were less common in non-muscle-invasive bladder cancers (20% of tumors) [16–18]. The protein UTX encoded by gene KDM6A has the function of catalyzing the demethylation of histone H3, and is involved in cell differentiation and tumor suppression [19]. However, there are no FDA-approved drugs targeting KDM6A, TP53 and other alteration genes except FGFR3. FGFR3-TACC3 is the only alteration which has clinical significance. It has been reported that the prevalence of FGFR3-TACC3 fusion in bladder cancer is 2–3% [20–22] and erdafitinib was the first orally effective FGFR antagonist approved by the FDA (2019) for the treatment of the urothelial carcinoma [23]. As our case occurred in the year of 2014, we choose pazopanib, of which FGFR3 was one of the target for the treatment. We found FGFR3-TACC3 fusion was not identified in the WES. As the detection of WES uses Agilent’s commercial WES capture probe kit, which mainly covers the coding region and extension sequence but not the intron regions. The breakpoint of FGFR fusion occurred in the intron of FGFR, which was not covered by the Agilent’s WES capture region, so the fusion of FGFR cannot be detected.

Like most complex genomic phenomena, acquired resistance to targeted therapies has diverse underlying drivers including both pathway-dependent mechanisms such as bypass activation, secondary mutation or downstream activation, and pathway-independent mechanisms such as tumor microenvironment, epithelial-mesenchymal transition (EMT) or epigenetic modulation [24]. Despite its role in tumorigenesis, the epigenetic modifications have been noticed to associate with acquired drug resistance in recent years [25]. And our analyses of genomic alterations pre- and post-pazopanib resistance suggested that epigenetic regulation might play a role in acquired TKI-resistance. The following 5 mutations were identified by all three screens (Panel1 and WES1 before TKI treatment, WES2 after drug resistance): EP300 Q224*, FAM135B L633*, KDM6A H900Qfs*11, IGF2 Q7P, TP53 E258K. Thus, those mutations represented bona fide early events in tumorigenesis, and they all have very high VAF. By contrast, the overwhelming majority of mutations arose post drug resistance and nearly all had very low VAF. The median VAF was 0.21 in the existed mutations pre-TKI resistance and 77.8%(7/9) mutations with a VAF higher than 10%, while the median VAF was 0.06 in newly detected mutations post TKI resistance and only 17.0%(9/53) mutations with a VAF higher than 10% (*p* = 2.682*10^− 7^). From the perspective of mutation abundance, drug-resistant mutations tend to be subclones, and these subclones mainly focus on epigenetic regulatory signaling pathways. Out of the new mutations arising post drug resistance, multiple genes from multiple epigenetic regulator families were hit, some multiple times. Strikingly, ARID1A and ARID1B saw no mutations before resistance but each had four separate mutations afterwards; similarly, KDM6A had only one mutation before resistance but saw three additional ones afterwards (Table 1). By carrying out functional prediction through bioinformatics prediction tool SNAP2 (Suppl Tab. 2), we found ARID1A/B both carry mutations predicted to alter protein function. ARID1A had 4 mutations, 2 of which were structural mutations at functionally important sites, while the other 2 were predicted to be functionally important. Out of the 4 mutations in ARID1B, 2 were predicted to be strongly functional (M479I, S397A). As ARID1A/B are mutually exclusive subunits of the same pathway, it implicates the SWI/SNF chromatin remodeling complex and epigenetic regulation as potential drivers of acquired TKI resistance. ARID1A and ARID1B belong to the AT-rich interactive-containing domain (ARID) gene superfamily [26]. ARID1A may play a tumor suppresser role, as studies have showed that increased proliferation and decreased apoptosis ensued after ARID1A knockdown [27–30]. It has been reported that ARID1A mutations tend to co-occur with CTNNB1 or PI3K-Akt pathway alterations, and are mutually exclusive with TP53 mutations [31]. Our observed ARID1A alterations despite the presence of TP53 mutations could reflect unique tumor features under drug pressure, and such unusual co-mutations could play a key role in acquired drug resistance. Another epigenetic function strongly implicated in TKI-resistance is histone methylation, with writer NSD1 mutated twice and eraser KDM6A mutated four times. As KDM6A works as a tumor suppressor gene [26], its malfunction could possibly contribute to drug resistance.

In addition, other interesting genetic mutations identified in tumor tissue at the time of pazopanib resistance included DICER1. At the first amino acid position of DICER1, methionine turned into leucine, which could lead to the deletion or abnormality of DICER1 protein. DICER1 serves several essential physiological functions including proliferation and survival cascades, and its abnormality might serve as another contributor to drug resistance [32, 33].

At the point of FGFR TKI-resistance, doubly mutated gene FANCD2 might be the driver of tumor re-sensitivity to the original cisplatin regimen. FANCD2, a member of the Fanconi Anemia (FA) protein family, plays a vital role in DNA damage repair [34]. Many studies have shown that mutations in DNA repair genes are important predictors of response to platinum drugs [35–37]; in particular, invasive bladder cancer with alterations in DNA damage repair related genes (ATM, RB1, FANCC) was found to be more sensitive to cisplatin-based chemotherapy [38]. WES finding of FANCD2 mutations might help explain the intriguing result of our PDX model, namely the possible re-sensitization of tumor cells to the original cisplatin regimen. Unfortunately, due to poor physical condition, the patient had no further opportunity to try cisplatin again. Recently, immune checkpoint inhibitors have triggered oncologists’ enthusiasm. PD-1/PD-L1 expression, tumor mutational burden, and DNA mismatch repair deficiency (dMMR) have been demonstrated as three potential biomarkers for immune checkpoint inhibitors [39–42]. Along tumor genetic alterations induced by drug pressure, significantly higher TMB was observed in this patient, suggesting possible utility of immunotherapy. In addition, ARID1A inactivation is associated with compromised MMR and increased mutagenesis, which might further cooperate with immune checkpoint blockade therapy [43].

In addition to the methods validated and insights obtained, the current study also had practical clinical implications. After the patient developed resistance to FGFR TKI, extensive epigenetic alterations raised the intriguing possibility of epigenetic inhibitor treatment, substantially elevated TMB suggested that the patient might be a good responder to immunotherapy, while possible (re)-sensitivity to cisplatin hinted at chemotherapy as a third-line option.

## Conclusions

Working alongside traditional wet-lab and clinical approaches, NGS technologies coupled with analytic tools from systems biology promise to reveal new insight into potential drug resistance mechanisms and epigenetic regulation. Our proof-of-concept study traced the tumor genetic variation course from chemo-resistant but TKI-sensitive to TKI-resistant but possible chemo-(re) sensitive, blending bench work and bioinformatics to obtain a better understanding of the complicated drug-driven mechanisms of resistance.

## Supplementary information

**Additional file 1: Figure S1.** Computed tomography (CT) imaging and Hematoxylin and eosin (H&E) staining of the patient. (a) Computed tomography (CT) imaging of the abdomen before the patient took gemcitabine and cisplatin regimen. (b) CT imaging of the abdomen after the patient took 6 cycles of gemcitabine and cisplatin regimen. (c) Hematoxylin and eosin (H&E) staining of patient tumor in segmental cystectomy. (d) Hematoxylin and eosin (H&E) staining of patient tumor after the patient got resistance to pazopanib.

**Additional file 2: Figure S2.** Tumorigenesis hubs are network centers. Red line marks the Closeness centrality of tumorigenesis genes (those mutated pre-resistance), while the histogram depicts the distribution of the same measure of 10,000 equivalent random samples, each of which has the same number of genes and the same degree distribution as the set of tumorigenesis genes. The fraction of random samples with Closeness centrality less than or equal to the red line was taken as the empirical *p*-value.

**Additional file 3: Figure S3.** Drug resistance hubs are more clustered. Red line marks the Clustering Coefficient (CC) of drug resistance genes (those mutated post-resistance), while the histogram depicts the distribution of the same measure of 10,000 equivalent random samples, each of which has the same number of genes and the same degree distribution as the set of drug resistance genes. The fraction of random samples with CC less than or equal to the red line was taken as the empirical p-value.

**Additional file 4: Figure S4.** Drug resistance hubs themselves connect hubs. Red line marks the K1 centrality of drug resistance genes (those mutated post-resistance), while the histogram depicts the distribution of the same measure of 10,000 equivalent random samples, each of which has the same number of genes and the same degree distribution as the set of drug resistance genes. The fraction of random samples with K1 centrality less than or equal to the red line was taken as the empirical p-value.

**Additional file 5: Figure S5.** GO enrichment analysis of drug resistance genes. GO-Slim terms were used to offer a simplified high-level overview, and all three GO categories were included: cellular component, biological process, molecular function. Color gradient corresponds to significance of corrected

**Additional file 6: Table S1.** Panel 1 gene list.

**Additional file 7: Table S2.** SNAP2 analysis to predict the protein function alteration of the mutations in post-TKI resistance.

## Data Availability

The raw datasets generated during the current study are not publicly available because it is possible that individual privacy could be compromised.

## Abbreviations

NGS: Next-generation sequencing
GP: Gemcitabine and cisplatin
PPI: Protein-protein interaction
CC: Clustering coefficient
GO: Gene Ontology
WES: Whole-exome sequencing
TKI: Tyrosine kinase inhibitor
PFS: Progression-free survival
PDX: Patient-derived xenograft
TMB: Tumor mutational burden
TGI: Tumor growth inhibition
