# Supplementary figures and images for: Whole-exome sequencing reveals potential mechanisms of drug resistance to FGFR3-TACC3 targeted therapy and subsequent drug selection: towards a personalized medicine
Source: BMC Med Genomics. 2020 Sep 21;13:138. doi: 10.1186/s12920-020-00794-x (PMC7507681; doi:10.1186/s12920-020-00794-x)

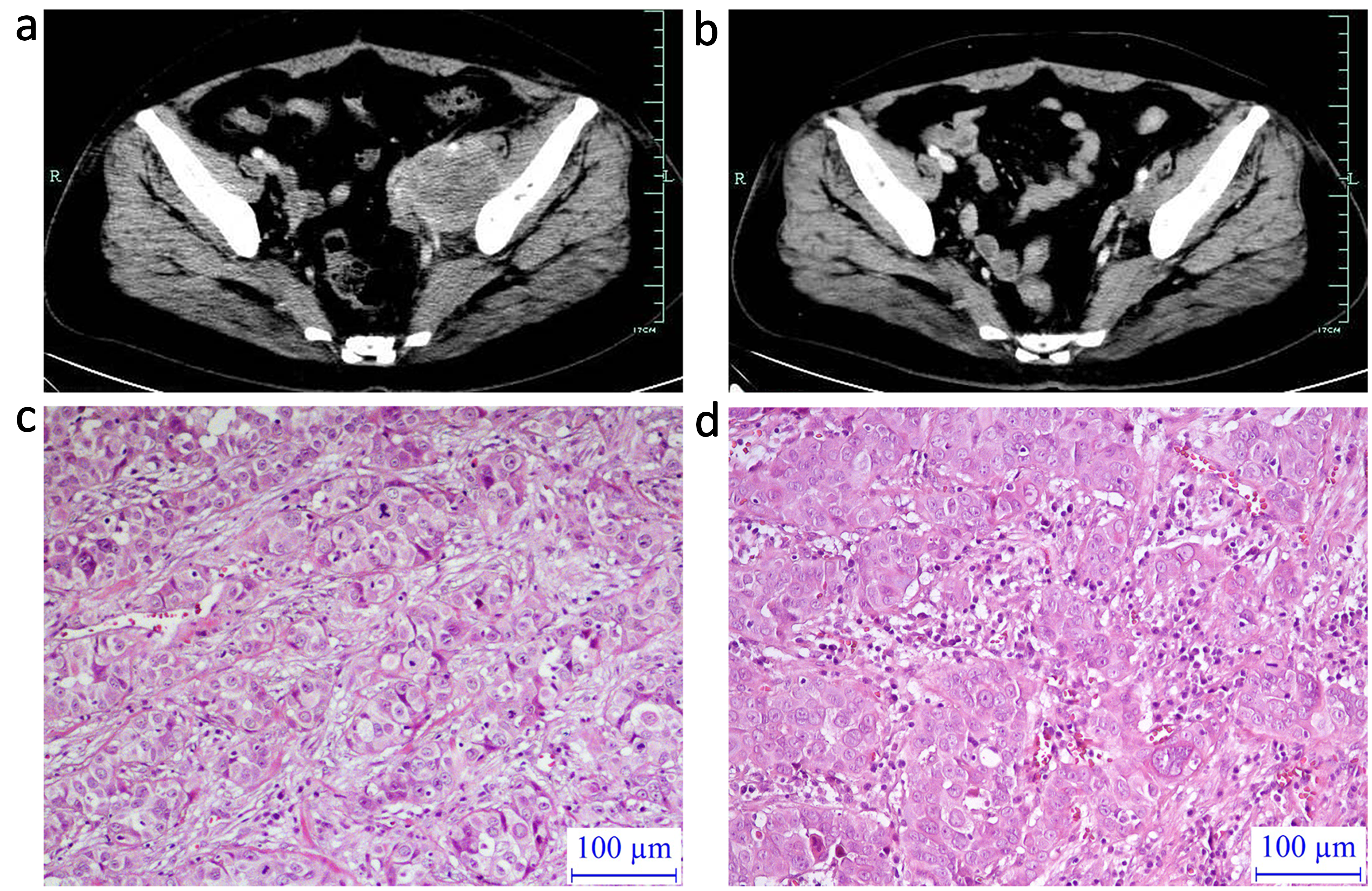

Supplement: Supplementary file 1 — Additional file 1: Figure S1. Computed tomography (CT) imaging and Hematoxylin and eosin (H&E) staining of the patient. (a) Computed tomography (CT) imaging of the abdomen before the patient took gemcitabine and cisplatin regimen. (b) CT imaging of the abdomen after the patient took 6 cycles of gemcitabine and cisplatin regimen. (c) Hematoxylin and eosin (H&E) staining of patient tumor in segmental cystectomy. (d) Hematoxylin and eosin (H&E) staining of patient tumor after the patient got resistance to pazopanib. [file 12920_2020_794_MOESM1_ESM.tif]

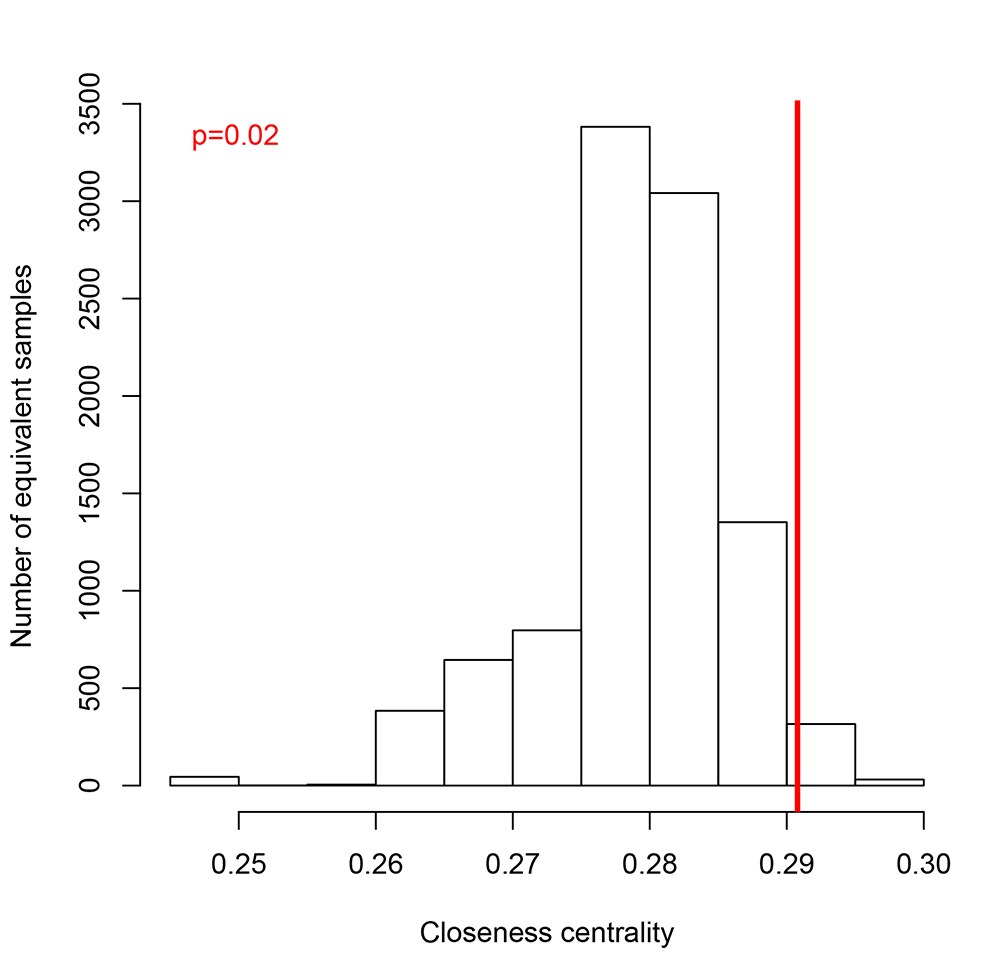

Supplement: Supplementary file 2 — Additional file 2: Figure S2. Tumorigenesis hubs are network centers. Red line marks the Closeness centrality of tumorigenesis genes (those mutated pre-resistance), while the histogram depicts the distribution of the same measure of 10,000 equivalent random samples, each of which has the same number of genes and the same degree distribution as the set of tumorigenesis genes. The fraction of random samples with Closeness centrality less than or equal to the red line was taken as the empirical p-value. [file 12920_2020_794_MOESM2_ESM.tif]

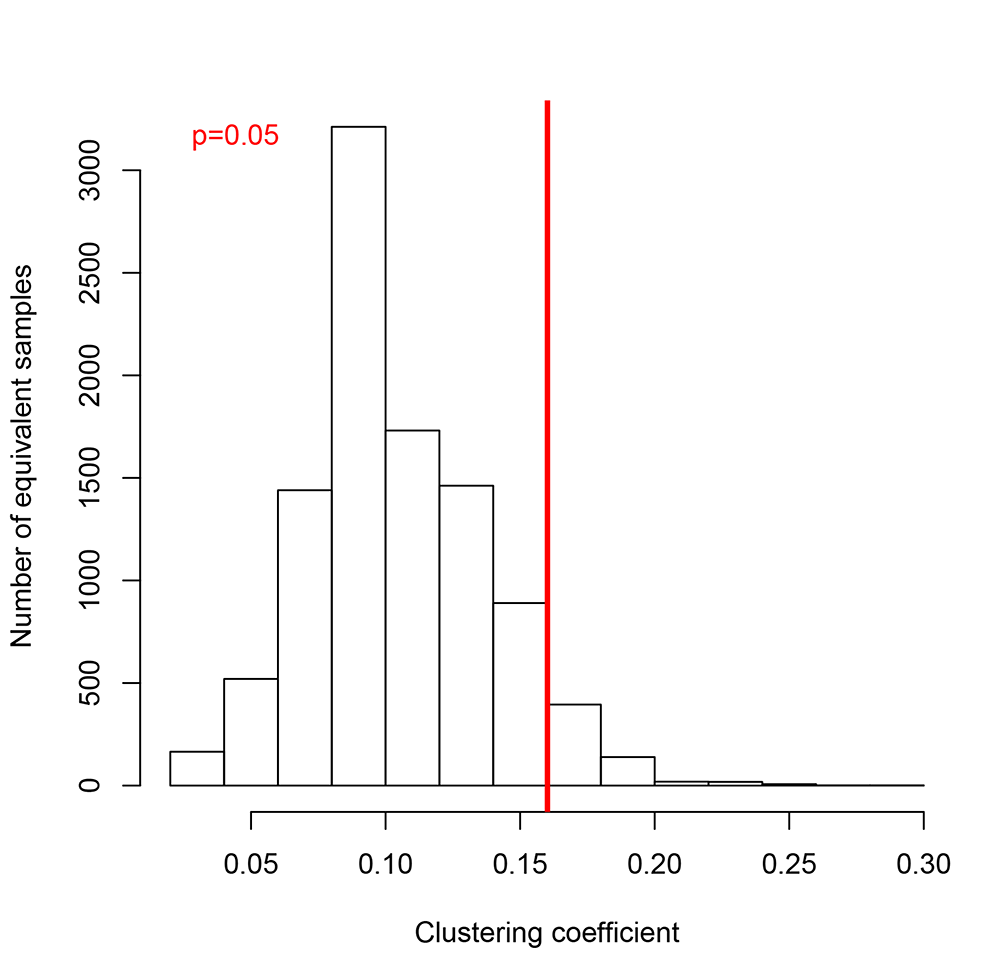

Supplement: Supplementary file 3 — Additional file 3: Figure S3. Drug resistance hubs are more clustered. Red line marks the Clustering Coefficient (CC) of drug resistance genes (those mutated post-resistance), while the histogram depicts the distribution of the same measure of 10,000 equivalent random samples, each of which has the same number of genes and the same degree distribution as the set of drug resistance genes. The fraction of random samples with CC less than or equal to the red line was taken as the empirical p-value. [file 12920_2020_794_MOESM3_ESM.tif]

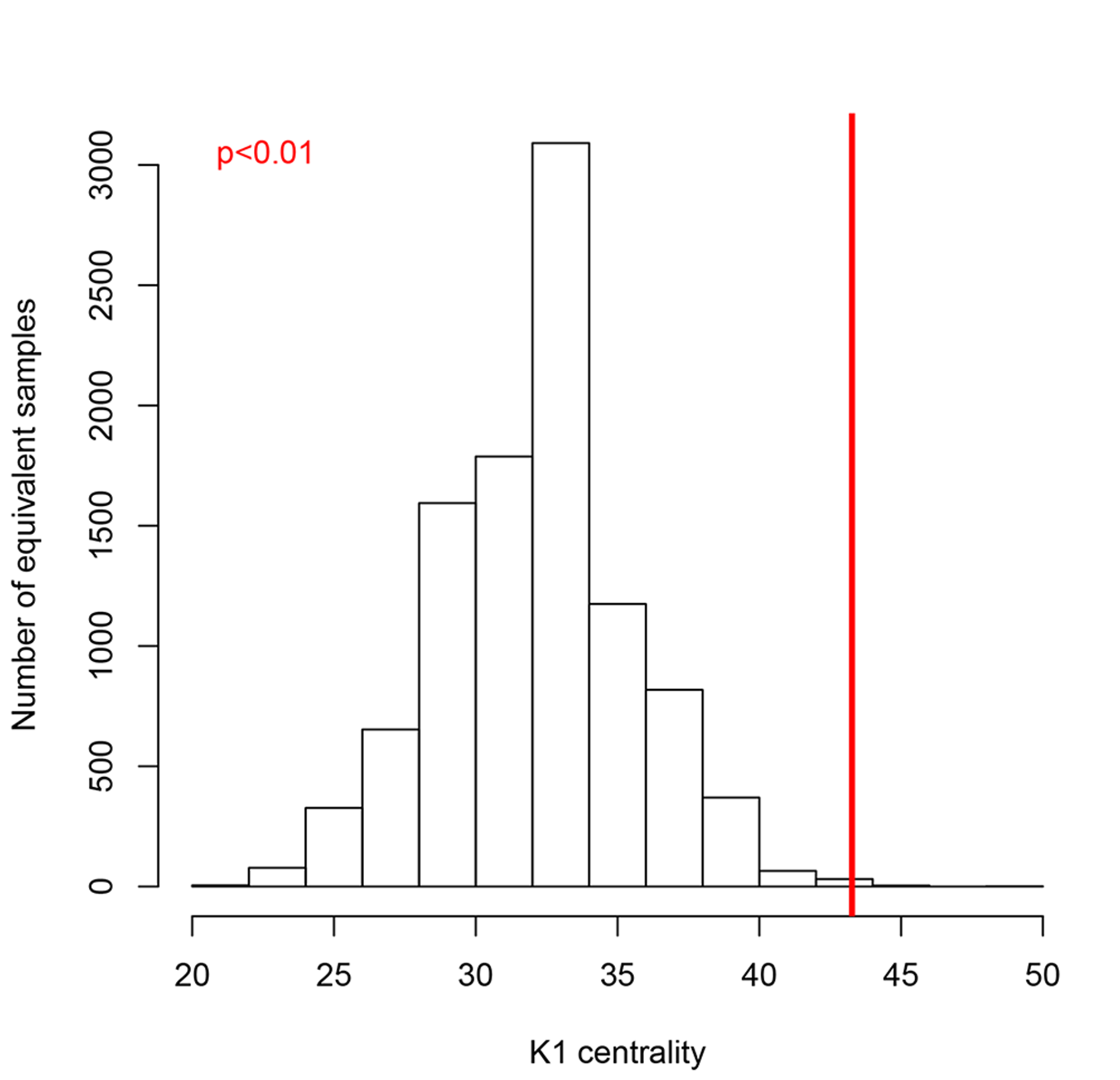

Supplement: Supplementary file 4 — Additional file 4: Figure S4. Drug resistance hubs themselves connect hubs. Red line marks the K1 centrality of drug resistance genes (those mutated post-resistance), while the histogram depicts the distribution of the same measure of 10,000 equivalent random samples, each of which has the same number of genes and the same degree distribution as the set of drug resistance genes. The fraction of random samples with K1 centrality less than or equal to the red line was taken as the empirical p-value. [file 12920_2020_794_MOESM4_ESM.tif]

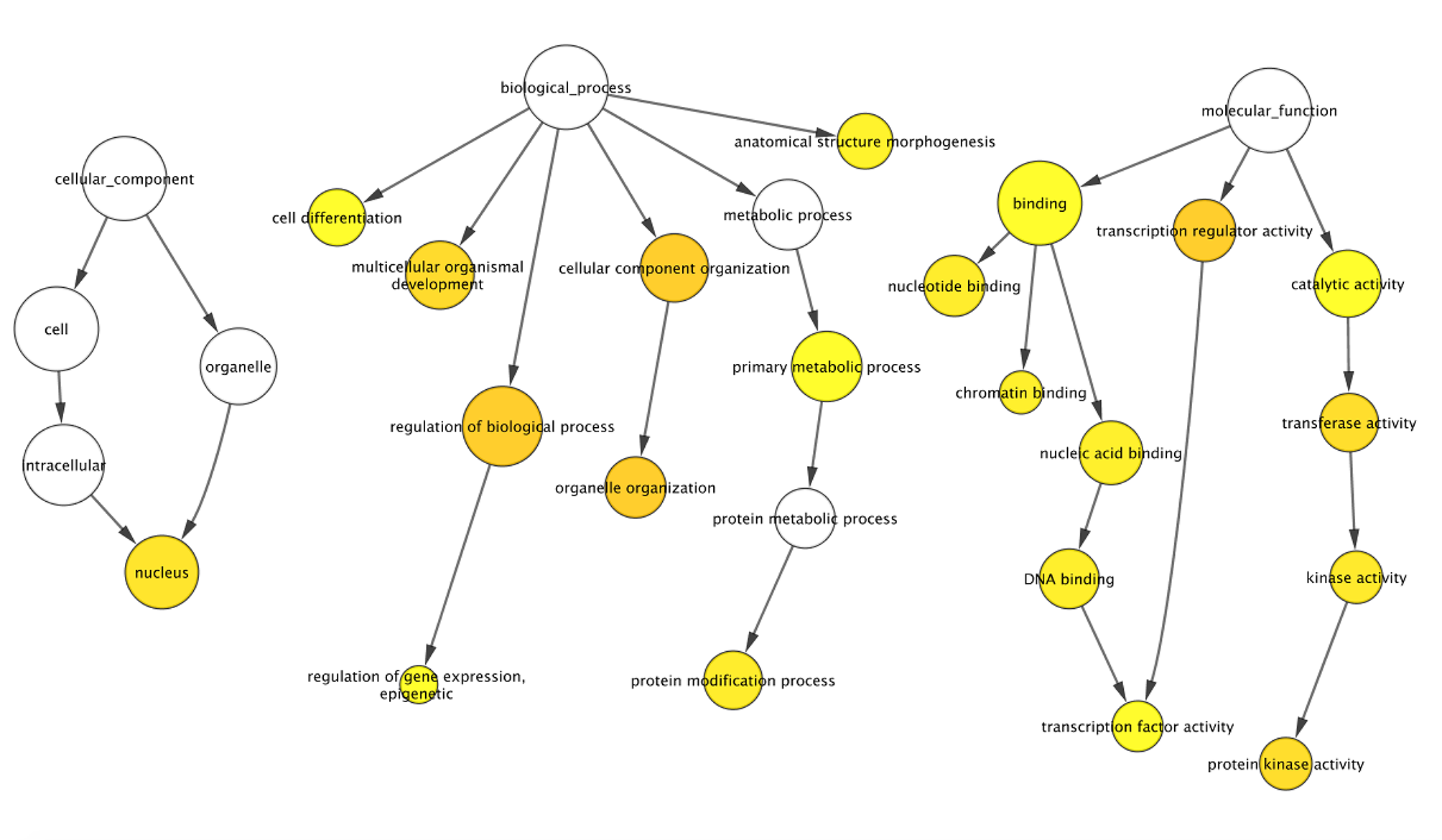

Supplement: Supplementary file 5 — Additional file 5: Figure S5. GO enrichment analysis of drug resistance genes. GO-Slim terms were used to offer a simplified high-level overview, and all three GO categories were included: cellular component, biological process, molecular function. Color gradient corresponds to significance of corrected [file 12920_2020_794_MOESM5_ESM.tif]
