# Supplementary material for: Whole-exome sequencing reveals potential mechanisms of drug resistance to FGFR3-TACC3 targeted therapy and subsequent drug selection: towards a personalized medicine
Source: BMC Med Genomics. 2020 Sep 21;13:138. doi: 10.1186/s12920-020-00794-x (PMC7507681; doi:10.1186/s12920-020-00794-x)
Supplement: Supplementary file 7 — Additional file 7: Table S2. SNAP2 analysis to predict the protein function alteration of the mutations in post-TKI resistance. [file 12920_2020_794_MOESM7_ESM.docx]

**Supplementary table 2. SNAP2 analysis to predict the protein function alteration of the mutations in post-TKI resistance.**

| Gene | Variation | Effect | Mean | Median | All Mean | All Median |
| --- | --- | --- | --- | --- | --- | --- |
| ACVR1B | V29I | -85 | -70.1 | -77 | 5.1 | 11 |
| ADGRA2 | S628C | -56 | -57.7 | -65.5 | 11.6 | 18 |
| AKT3 | T144T | -88 | 3.75 | 13.5 | 12.2 | 22 |
| ARHGAP26 | R719W | 76 | 42.45 | 46.5 | 13.5 | 23 |
| ARID1A | T118P | 16 | 3.75 | 15 | 27.4 | 34 |
| ARID1A | P153A | 22 | 26.25 | 31.5 | 27.4 | 34 |
| ARID1A | S261- | 17.3 | 17.3 | 23.5 | 27.4 | 34 |
| ARID1A | A347$ | 45.1 | 45.1 | 57.5 | 27.4 | 34 |
| ARID1B | G278S | -32 | -11.65 | -10 | -8.0 | -7 |
| ARID1B | C295Y | -34 | -43.45 | -41 | -8.0 | -7 |
| ARID1B | M479I | 53 | 25.2 | 31.5 | -8.0 | -7 |
| ARID1B | S397A | 49 | 51.8 | 61 | -8.0 | -7 |
| CDK12 | A497S | -72 | -42.55 | -41 | 9.9 | 12 |
| CDK6 | M126I | -60 | 2.35 | 32 | 5.5 | 12 |
| CDK8 | H235Y | -62 | -46.6 | -54 | 11.8 | 15 |
| CHD4 | M76L | -33 | -28.4 | -26 | 17.0 | 24 |
| CREBBP | G49- | 13.05 | 13.05 | 24 | 31.5 | 36 |
| CSNK1A1 | T169P | 34 | 11.9 | 25.5 | 16.3 | 31 |
| CUL3 | D155V | 49 | 30 | 50.5 | 0.7 | 10 |
| DICER1 | M1L | -21 | 37.05 | 49 | 1.5 | 5 |
| DPYD | N57S | -92 | -77.35 | -80 | 2.2 | 12 |
| DPYD | M77V | -49 | -46.3 | -54 | 2.2 | 12 |
| EP300 | Q224* | -50.8 | -50.8 | -47.5 | -7.6 | -6 |
| EPHA5 | E601D | -43 | -36.75 | -34 | 4.0 | 13 |
| ERBB3 | E881Q | 71 | 69.25 | 77 | 10.2 | 14 |
| ETV1 | N23T | -71 | -50.9 | -46.5 | 18.9 | 23 |
| EWSR1 | M300L | 31 | 48.5 | 50 | 32.8 | 38 |
| EWSR1 | M330L | 28 | 41.9 | 45 | 32.8 | 38 |
| EXT1 | V292V | -93 | -60.8 | -61.5 | 3.9 | 12 |
| EXT1 | V293L | -3 | -0.95 | 4 | 3.9 | 12 |
| FAM135B | L633* | -26.65 | -26.65 | -21 | 10.9 | 14 |
| FANCD2 | S240* | -37.5 | -37.5 | -45 | 19.7 | 32 |
| FANCD2 | H287Y | 41 | 14.85 | 23 | 19.7 | 32 |
| FAT3 | P2285H | -39 | -25 | -28 | -7.4 | -5 |
| FLT1 | G839* | 74.85 | 74.85 | 83.5 | 0.2 | 3 |
| FOXP1 | K542I | -19 | -19.85 | -11.5 | -10.1 | -9 |
| GLI2 | P1371L | 61 | 52.45 | 61 | 22.2 | 30 |
| IGF2 | Q7P | 57 | 28.7 | 33 | 28.1 | 35 |
| IKZF1 | D116H | 68 | 61.6 | 67 | 48.5 | 55 |
| KAT6A | G1590V | -23 | -3.25 | 0.5 | 7.1 | 9 |
| KDM5A | M132I | -18 | -10.6 | -13.5 | 13.2 | 19 |
| KDM6A | H900& | -28.6 | -28.6 | -29 | 14.7 | 22 |
| KDM6A | I776T | -69 | -30.45 | -31.5 | 14.7 | 22 |
| KDM6A | S475P | 24 | 17.65 | 24.5 | 14.7 | 22 |
| KDM6A | A680T | -48 | -19.6 | -13 | 14.7 | 22 |
| NCOR1 | T1870N | -54 | -27.9 | -29.5 | 8.0 | 12 |
| NF1 | K1385R | 23 | 29.45 | 31 | 10.5 | 19 |
| NFIB | S449T | -77 | -15.85 | -13 | 17.7 | 22 |
| NKX2-1 | S6G | 28 | 13.3 | 19.5 | 23.9 | 29 |
| NRG3 | E355D | -14 | 25.05 | 34.5 | 10.1 | 14 |
| NSD1 | T52^ | 20.6 | 20.6 | 26.5 | 11.4 | 15 |
| NSD1 | V53A | -11 | 53.05 | 69 | 11.4 | 15 |
| NTRK3 | P541S | -76 | -54.25 | -65 | -3.2 | 2 |
| PLAG1 | V18A | 28 | 13.3 | 28 | 12.8 | 17 |
| RANBP2 | D1157H | 35 | 19.85 | 31 | 19.0 | 24 |
| RHOA | E186K | -42 | -18.6 | -18 | 34.3 | 46 |
| SMARCA4 | A1423A | -99 | -7.45 | -4 | 27.1 | 36 |
| SSX1 | K153N | 37 | 31.6 | 31.5 | 32.6 | 36 |
| TCF7L2 | S467A | 30 | 25.75 | 33.5 | 17.1 | 23 |
| TEK | S1084* | -12.35 | -12.35 | -1 | 9.2 | 14 |
| TERT | E507V | 26 | 25.05 | 32 | 11.1 | 21 |
| TP53 | E258K | 79 | 57.5 | 68 | 4.6 | 7 |
| ZNF703 | G21^ | -8.25 | -8.25 | -10 | 17.5 | 23 |

Notes: Column 1 is the gene name; Column 2 is the observed mutation on the canonical protein sequence; Column 3 is the prediction score of the given mutation impacting protein function; Columns 4 and 5 are the mean and median scores of all possible mutations at this site (changing the native amino acid into all other possible ones); Columns 6 and 7 are the mean and median scores of all possible mutations at all sites for this protein sequence.

The estimated effect has a score between -100 and 100, with scores >50 considered “strong effect”, i.e. highly likely impacting protein function; with scores <-50 considered “strong neutral”, i.e. highly likely not impacting function; scores from 0 to 50 are “weak effect”, from -50 to 0 “weak neutral”.
